# Management of Dysarthria in Amyotrophic Lateral Sclerosis

**DOI:** 10.3390/cells14141048

**Published:** 2025-07-09

**Authors:** Elena Pasqualucci, Diletta Angeletti, Pamela Rosso, Elena Fico, Federica Zoccali, Paola Tirassa, Armando De Virgilio, Marco de Vincentiis, Cinzia Severini

**Affiliations:** 1 Department of Sense Organs, Sapienza University of Rome, 00161 Rome, Italy; elenapasqualucci@yahoo.it (E.P.); diletta.angeletti@uniroma1.it (D.A.); federica.zoccali@uniroma1.it (F.Z.); armando.devirgilio@uniroma1.it (A.D.V.); marco.devincentiis@uniroma1.it (M.d.V.); 2 Insitute of Biochemistry and Cell Biology, National Research Council of Italy, 00161 Rome, Italy; pam.rosso@gmail.com (P.R.); elena.fico@ibbc.cnr.it (E.F.); paola.tirassa@cnr.it (P.T.)

**Keywords:** amyotrophic lateral sclerosis (ALS), dysarthria, diagnosis, speech biomarkers, speech therapy

## Abstract

Amyotrophic lateral sclerosis (ALS) stands as the leading neurodegenerative disorder affecting the motor system. One of the hallmarks of ALS, especially its bulbar form, is dysarthria, which significantly impairs the quality of life of ALS patients. This review provides a comprehensive overview of the current knowledge on the clinical manifestations, diagnostic differentiation, underlying mechanisms, diagnostic tools, and therapeutic strategies for the treatment of dysarthria in ALS. We update on the most promising digital speech biomarkers of ALS that are critical for early and differential diagnosis. Advances in artificial intelligence and digital speech processing have transformed the analysis of speech patterns, and offer the opportunity to start therapy early to improve vocal function, as speech rate appears to decline significantly before the diagnosis of ALS is confirmed. In addition, we discuss the impact of interventions that can improve vocal function and quality of life for patients, such as compensatory speech techniques, surgical options, improving lung function and respiratory muscle strength, and percutaneous dilated tracheostomy, possibly with adjunctive therapies to treat respiratory insufficiency, and finally assistive devices for alternative communication.

## 1. Introduction

Amyotrophic lateral sclerosis (ALS) is a rare neurodegenerative disease characterized by the selective degeneration of motor neurons (MNs) with an extensive clinical phenotype, progressive development, and an unfavorable prognosis [1]. It affects both upper motor neurons, which extend from the cortex to the brainstem and spinal cord, and lower motor neurons, which connect the brainstem or spinal cord to the muscles [2]. Respiratory muscle paralysis is the main cause of death in ALS patients and occurs within 3 to 5 years of diagnosis, although in a small percentage of patients, the survival time is much longer, around 10 years [3].

### 1.1. Epidemiology

ALS is a globally prevalent neuromuscular disorder, affecting individuals across various ethnic and demographic backgrounds. The majority of cases—over 90%—are sporadic (sALS), appearing without clear genetic links or identifiable risk factors.

A smaller proportion, approximately 5–10%, represents familial ALS (fALS). They are due to mutation in the genes directly linked to motor neuron degeneration, including a defect in a gene known as “chromosome 9 open reading frame 72” or *C9ORF72*. The mutation of this gene is responsible not only for motor symptoms but also for cognitive impairment and dementia. Other familial cases are characterized by a mutation in the gene that encodes for the production of the enzyme copper–zinc superoxide dismutase 1 (*SOD1*) [4]. The two forms are clinically and pathologically similar, suggesting a common pathogenesis [5].

Among the potential risk factors for ALS, advanced age appears to play a role, as the incidence increases with age, reaching a peak around the age of 60. In addition, gender differences in the onset of the disease are evident, with an overall male to female ratio of 1.35, which is influenced by the age of onset. In terms of ethnicity, Caucasians appear to be more likely to develop the disease. Another risk factor is persistent organic pollutants and metals in the blood, which increase the risk of disease and shorten survival. One category twice as likely to develop the disease is war veterans, probably due to exposure to toxic substances such as pesticides [3].

### 1.2. Etiopathogenesis

Although the pathogenesis of ALS is not fully understood, several mechanisms have been identified that may be involved in the degeneration of MNs. A central feature of ALS is mitochondrial dysfunction [6] and the resulting increased oxidative stress [7].

Additional mechanisms include impaired glutamate uptake by glial cells [8], neuronal hyperexcitability and excitotoxicity due to glutamate overactivity [9], systemic hypermetabolism [10], and chronic neuroinflammation [11]. Disruptions in intracellular transport, DNA repair errors, and imbalances in protein folding and degradation have also been implicated in the disease process [12].

A key finding in both familial and sporadic ALS is the accumulation of misfolded SOD1 proteins, which appear capable of spreading between cells in a prion-like manner [13], confirming data observed in other neurodegenerative diseases, e.g., Parkinson’s disease (PD) [14]. Indeed, it has been proposed that the α-synuclein pathology in PD begins in the intestinal nervous system due to gut microbiota dysbiosis, infection, and inflammation, and subsequently spreads from the gastrointestinal tract into the CNS via the vagus nerve and reaches the lower brainstem via retrograde axonal transport [15].

### 1.3. Diagnosis and Therapy

ALS diagnosis and classification are still based on clinical criteria and functional scales or staging systems, such as the ALS Functional Rating Scale-Revised (ALSFRS-R), which aim to measure disease progression [16]. The diagnostic process is often prolonged—sometimes taking up to a year—and typically includes clinical evaluations, neurophysiological studies, and imaging techniques. A significant challenge in early and accurate diagnosis is the absence of definitive biological markers. Laboratory tests may be necessary to rule out alternative diagnoses. Biomarkers like neurofilament light chain (NfL) and phosphorylated neurofilament heavy chain in cerebrospinal fluid and serum have shown moderate correlations with disease progression, yet they are not routinely incorporated into standard diagnostics [2]. Genetic screening, now more accessible in industrialized nations, helps identify the mutations linked to increased ALS susceptibility or causation [17].

The most common symptoms are the progressive weakness of the limbs, neuromuscular respiratory failure and dysarthria, and dysphagia. Other clues pointing to a diagnosis of ALS include unexplained weight loss, pseudobulbar affect, changes in cognitive abilities or executive functions, and a family history of ALS or other neurodegenerative diseases. The clinical history and neurological examination are accompanied by serological and electrodiagnostic tests such as electromyography (EMG) and nerve conduction studies (NCSs) [3]. Early detection is essential, not only for eligibility in clinical trials but also because it allows for timely medical interventions that can prolong survival and improve quality of life [18].

There is currently no real treatment for ALS; management is focused on using disease-modifying therapies and maximizing quality of life. Drug discovery for ALS has relied on traditional approaches with limited success. Despite more than a hundred phase II and phase III trials in the decade to 2019, effective disease-modifying drugs remain scarce [19]. The negative results of these studies could be due to the clinical and pathogenic heterogeneity of the disease and errors in trial design [20]. Only the anti-glutamate agent riluzole (Rilutek), which was developed for ALS almost 3 decades ago, is generally approved for the disease and prolongs survival by 6 to 19 months, especially in the bulbar form of the disease [21]. Treatment with edaravone, an antioxidant drug approved by the FDA to treat ALS patients [22], also showed some efficacy in slowing the progression of the disease, although its use remains controversial [23].

The two most recent FDA-approved drugs, Relyvrio (sodium phenylbutyrate and taurursodiol) and Tofersen (Qalsody), have completed trials and shown that they slow the progression of the disease by a few months in a selected population. However, they do not stop the progression of the disease. It is worth noting that Relyvrio is not currently available for treatment due to the negative results of the Phenix phase III trial [24,25].

The difficulty of early diagnosis and the incompletely understood mechanism of the selective and progressive degeneration of motor neurons limit the identification of effective treatments and remain a challenge for ALS research. The treatment of ALS focuses on multidisciplinary care to control symptoms such as muscle spasms, rigidity, excessive saliva and mucus, and pseudobulbar affect, and ultimately to support end-of-life planning.

The most common drugs containing riluzole can slow the progression of the disease and reduce the deterioration of everyday functions. The therapeutic strategies may be more effective if the disease is diagnosed at an early stage and subsequently treated early [26].

### 1.4. ALS Classification

The classification of ALS may vary depending on the criteria used. The traditional definitions of ALS subgroups are based on the extent of upper and lower motor neuron involvement, although other classification systems include other parameters, such as the site of onset (i.e., bulbar or spinal onset of the disease). “Limb onset” of ALS is indicated when symptoms begin in the arms or legs, with muscle weakness or stiffness, followed by the gradual impairment of all voluntary muscles, including the muscles responsible for breathing [3]. In other patients, referred to as “bulbar onset” ALS, the first symptoms are swallowing disorders (dysphagia), shortness of breath (dyspnea), or speech and word formation disorders (dysarthria) [27]. A subset of patients may exhibit language or decision making disorders, with increasing evidence of the occurrence of dementia in some cases [28].

Physiological impairments in the orofacial muscles among ALS patients result in compromised speech and communication, with diminished control over the larynx, lips, tongue, jaw, and voice box [29,30,31]. Slowed articulation speed may serve as one of the earliest indicators of tongue motor neuron degeneration linked to ALS-related dysarthria [32]. Up to 93% of people with bulbar disease suffer from dysarthria, with upper and lower motor neuron involvement leading to mixed dysarthria with flaccid and spastic features as the disease progresses [27]. A reduced rate of speech is frequently among the earliest signs of dysarthria and serves as a predictive marker for the subsequent decline in verbal communication [33].

In this review, we examine the mechanism underlying the language disorder. We discuss the most promising digital speech biomarkers of ALS that are important for early diagnosis, as well as the impact of interventions that can improve voice function and patient quality of life.

## 2. ALS Dysarthria

Speech is an intricate, high-speed motor function requiring precise coordination among numerous anatomical structures. It blends voluntary motor control with reflexive actions, all influenced by cognitive and emotional inputs.

Basically, the voice involves the precise coordination of more than 100 laryngeal, orofacial, and respiratory muscles, controlled by the motor and sensory nuclei of the lower brain stem and the spinal cord. These are interconnected and coordinated by the nuclei of the lateral reticular formation [34]. Together, they provide the basic control of laryngeal, articulatory, and respiratory activity [35]. The precise coordination required for human speech relies heavily on cortical control. Modern neuroimaging studies, particularly those using functional magnetic resonance imaging (fMRI), suggest that complex speech functions are governed by distributed neural networks rather than localized brain regions. These networks involve the cortical and subcortical motor areas, as well as bilateral temporal lobes, with a critical role attributed to the posterior temporal gyrus [36].

The central executive of this network is in the ventrolateral prefrontal cortex. At the same time, the basal ganglia and cerebellum play essential roles in language preparation, execution, and the acquisition of motor skills. Furthermore, the complex sensorimotor coordination and modulation of learned vocalizations for speech production result from the strong structural connectivity of the laryngeal motor cortex with the somatosensory and inferior parietal cortex [37].

Cortical thinning is well documented in people with ALS, and significant correlations between the impairment of specific cognitive processes and speech rate and neurostructural changes in different regions of the frontal lobe have been demonstrated [38,39]. In ALS, the bulbar deficit was previously associated with cortical thinning in the ventral precentral gyrus (motor) but not in the extramotor regions, supporting the hypothesis of an association of speech rate abnormalities with spastic dysarthric (motor) deficits in ALS and not with a cognitive deficit [40]. A study by Zaninotto et al. [39] further demonstrated that patients with functional speech impairments exhibited significant cortical thinning in key motor and sensory regions. Using high-resolution 3T MRI and both global and targeted brain analyses, they observed pronounced thinning in the left precentral and postcentral gyri and the right inferior parietal lobe, along with the adjacent lateral occipital cortical regions. These areas are crucial for controlling the orofacial structures involved in speech. The findings suggest that speech rate may be a sensitive indicator of cortical degeneration in ALS. Interestingly, the evaluated patterns of cortical thickness in a limited number of ALS patients with and without functional language changes suggest that speech rate may discriminate between patients with and without cortical thinning.

Individuals with ALS often experience reduced speech rapidity, altered vocal quality, and impaired articulation, despite retaining intelligibility in the early stages [41]. Functional deficits include diminished control over the larynx, lips, tongue, and jaw [42], along with slower tongue movements and disrupted coordination between different parts of the tongue during speech production [43]. This decline in articulation speed is considered one of the earliest signs of tongue motor neuron degeneration [32]. Before speech clarity deteriorates, changes in articulation, especially rapid movements, become evident. Ultimately, a progressive, mixed dysarthria—combining spastic and flaccid elements—emerges as a characteristic of ALS speech impairment [29].

As previously suggested, voice and speech assessment appears to be an effective method for diagnosing the disease in its clinical or preclinical state and monitoring its progression [42,44]. Over time, 80–95% of individuals with ALS lose their ability to communicate naturally, and many eventually become non-verbal [45]. For them, communication support includes a range of augmentative and alternative communication (AAC) strategies, including both simple and high-tech options (such as speech-generating devices) [46].

It has been shown that the majority of patients with suspected or possible ALS with bulbar onset have a poorer prognosis than patients with spinal onset, and are more likely to have a delayed diagnosis and delayed speech therapy assessment, emphasizing the importance of early treatment. For these patients, it is important to begin therapy to improve vocal function during the diagnostic process, as speech rate appears to decline significantly before the diagnosis of ALS is confirmed [42].

### Digital Speech Biomarkers

A new frontier in characterizing speech features is the analysis of digital speech biomarkers.

Given the complexity of dysarthria in ALS, acoustic analysis using digital tools offers a promising approach to identifying distinct and quantifiable speech features. By recording a patient’s vocal output via microphones, clinicians can capture acoustic data and convert it into digital signals that reveal abnormalities such as breathy or raspy voice quality, inconsistent vowels, monotony, low volume, and extended pauses [47]. Acoustic speech analysis is affordable and noninvasive, and recordings can be captured remotely using standard smartphone microphones. This enables clinicians to routinely evaluate speech in the clinic or during patients’ daily lives [48,49]. Automated speech analysis has already proven valuable in diseases like Parkinson’s (PD), where it has enhanced diagnostic precision and the enabled real-time monitoring of disease progression and therapeutic effects [50].

Moreover, while some speech biomarkers are shared across various neurological conditions, certain features can help distinguish between disorders with overlapping symptoms. For instance, in ALS, reduced speech rate and increased pause duration are common but not exclusive markers, potentially reflecting underlying cognitive impairment. Conversely, parameters like articulation rate and loudness often change in opposing directions across different conditions [50].

Table 1 (adapted from Supplementary Table 3 by Rusz et al. [50]), provides a detailed overview of the applicability of different speech biomarkers in ALS, emphasizing their diagnostic and prognostic utility.

## 3. Management of Dysarthria

Communication disorders are a significant barrier to activity and participation in social and civic life, negatively impacting quality of life. Dysarthria does not have to be severe to have a significant psychosocial impact on people’s lives.

Speech and breathing are closely linked: reduced phonatory function (e.g., hypo- or hyper-adduction of the vocal folds), insufficient respiratory support due to weak respiratory muscles, and/or reduced respiratory and phonatory control (difficulties in coordinating speech and breathing) negatively impact speech production in ALS [58,59]. Initial changes typically include altered voice quality, breathlessness, roughness, hoarseness, tension, and reduced volume [60,61].

ALS patients with mild or moderate dysarthria can benefit from compensatory speech techniques, where various techniques can improve poor speech comprehension and maintain communication skills for as long as possible [29]. Although exercises to strengthen the oropharyngeal muscles have been shown to be ineffective [62,63], various types of interventions appear to improve vocal function.

### 3.1. Speech Therapy

Specialized speech and language therapy to maintain communication has proven useful for ALS patients with mild to moderate dysarthria followed by the prescription of a communication device in advanced stages [27,64]. Speech therapy can contribute in particular to correcting the compensation strategies that are ineffective in patients with ALS. Indeed, the voice becomes breathy and weak when the vagus nerve is damaged, and the resonance becomes hypernasal when the muscles and nerves innervating the soft palate are affected [65]. Respiratory weakness (particularly due to damage to the diaphragm by the phrenic nerve) contributes to a soft voice as breathing becomes less supportive of speech [66].

In the initial phase of speech therapy, the focus is on clear, exaggerated articulation and compensatory techniques to make the voice audible, as increased effort can be counterproductive. To make themselves understood, patients tend to force their voice and articulate too much. Given progressive respiratory distress, breathing and relaxation exercises are useful to improve vocal skills. In speech therapy, patients are encouraged to use their voice and articulation patterns and breathing rate as sparingly as possible during a speech to optimize the use of available breathing resources. Voice amplifiers can also be helpful in this respect [65]. In addition, techniques such as vibration or the application of ice to the affected muscles can reduce the excessive muscle tone and thus affect the dysarthria [67].

### 3.2. Palatal Lift

Palatal lift for velopharyngeal dysfunction has been shown to increase speech intelligibility. Twenty-one patients (84%) treated with a palatal lift showed improvement in their dysarthria, particularly a reduction in hypernasality, with nineteen (76%) benefiting at least moderately for 6 months [68].

### 3.3. Pulmonary Function and Respiratory Muscle Strength

As the pharyngeal, laryngeal, and respiratory muscles work together during breathing to maintain upper airway patency and control airflow throughout the respiratory cycle [69], improving lung function and respiratory muscle strength can ameliorate dysarthria.

In motor neuron diseases, the respiratory muscles are affected, which manifests itself in the form of muscle atrophy and contractures and leads to restricted movement of the chest. This progressive constriction of the chest leads to a continuous decrease in vital capacity. Indeed, the degree of lung volume restriction is the strongest predictor of mortality in patients with ALS. In addition to inspiratory muscle failure, the progressive loss of lung volume and the weakness of expiratory muscles lead to cough failure, resulting in the impaired clearance of airway secretions, recurrent respiratory infections, and atelectasis [3]. Cough flow can be increased by mechanical insufflation techniques or by mechanical insufflation/exsufflation [70].

The effects of various forms of exercise on the function of patients with amyotrophic lateral sclerosis (ALS) have been the subject of discussions and studies for many years. The effectiveness of exercise and breathing training in ALS is limited and often controversial. In the past, respiratory training programs aimed at strengthening and mobility of the motor speech apparatus were considered contraindicated. However, clinical studies suggest a potential benefit of training respiratory function and respiratory muscle strength with a variety of breathing exercises [71].

Manually assisted coughing and mechanical insufflation–exsufflation, which combines an increase in inspiratory capacity with an externally applied negative pressure, have been used as non-invasive aids to assist coughing in bulbar and non-bulbar ALS patients. Negative pressure applied during coughing has been shown to increase transpulmonary pressure and expiratory cough flow [72]. The breath stacking technique (using a manual bag to recruit lung volume) [73] should be recommended as a first line intervention for lung recruitment and cough augmentation in ALS patients [74]. A retrospective study has shown that the use of a home protocol with the breath stacking technique can significantly reduce the hospitalization rate for respiratory complications in neuromuscular diseases, including ALS [75].

In addition, a randomized controlled trial has shown that non-invasive ventilation improves survival and maintains or improves quality of life in patients with ALS without severe bulbar dysfunction. The survival benefit of non-invasive ventilation in this group is much greater than that of currently available neuroprotective therapy. In patients with severe bulbar impairment, non-invasive ventilation improves sleep-related symptoms but is unlikely to provide a significant survival benefit [76,77].

Moreover, muscle training over an 8-week period with a targeted exercise intervention has been shown to improve expiratory muscle function [78]. Recently, a randomized controlled trial was conducted using breath stacking and expiratory muscle training (EMT) to improve airway function and support coughing in ALS patients. The preliminary results indicate that patients undergoing breath stacking plus EMT show a slower deterioration after 6 months than patients undergoing breath stacking alone [79].

### 3.4. Percutaneous Dilatational Tracheostomy (PDT)

When noninvasive ventilation techniques are no longer effective in treating symptoms, a percutaneous dilatational tracheostomy (PDT) can be performed to treat respiratory failure. This technique can extend patients’ lives [80]. In addition, PDT can preserve vocal function. Techniques to prolong inspiratory time and positive end-expiratory pressure in conjunction with the use of speaking tracheostomy tubes enable improved phonation, which has a significant impact on patients’ quality of life [81,82].

### 3.5. Percutaneous Dilatational Tracheostomy (PDT) and Adjuvant Therapies

Recent studies highlight the benefits of combining PDT with complementary therapies to further support vocal function in ALS. A retrospective analysis conducted four weeks after PDT showed that therapies such as respiratory and swallowing rehabilitation, regenerative injections, low-frequency electrical stimulation of the neck, and balloon dilatation protocols can collectively aid in restoring vocal abilities in ALS patients [83].

### 3.6. Aids for Alternative Communication

Since speech rate is a relatively good predictor of the deterioration in intelligibility in patients with spinal, bulbar, or mixed ALS [33], it is necessary to start providing aids for alternative communication in good time if the dysarthria progresses and the ALS patient’s speech rate falls below 100 words per minute [84].

Alternative means of communication range from visual or gestural cues to more sophisticated computer-based technology that “speaks” for the patient to improve communication skills. Examples include eye-tracking devices for answering yes–no questions, laser pointers attached to glasses or headbands used with letter boards, and computer systems equipped with speech synthesis activated via head or eye movements [65,66].

Some people with ALS opt for voice banking while they can still speak. This allows them to store their own voice (voice banking) for future use in computer-based speech synthesizers. Patients can be sent to record their voice patterns before they develop severe dysarthria so that the communication device can be programmed with their own voice instead of a robotic sound [85]. Advancements in artificial intelligence (AI) have further refined this process. By integrating voice banking with AI technologies, it is now possible to create synthetic voices with remarkable emotional expression and acoustic fidelity, offering a more personalized and natural communication experience for ALS patients [86].

## 4. Conclusions and Future Directions

**Figure 1:**
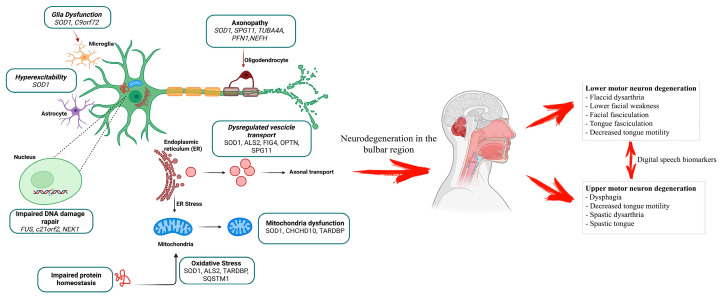
Clinical signs of bulbar degeneration affecting speech. Degeneration of the MNs involves multiple neural pathways. Degeneration in the bulbar region of the brainstem leads to symptoms affecting muscles of the face, pharynx, and tongue. This picture is created with BioRender.com.

Speech impairment (dysarthria) generally occurs earlier in bulbar ALS than in limb ALS. Nevertheless, all ALS patients eventually show speech deficits, which may be the first manifestation of the pathology. People with bulbar ALS initially have motor dysfunction in the bulbar region of the brainstem, leading to speech difficulties that are often accompanied by concomitant hypersalivation. As shown in Figure 1, the degeneration of the MNs at the bulbar level leads to the dysfunction of the muscles in the face, pharynx and tongue. There are different forms of dysarthria affecting the upper motor neuron (UMN) or the lower motor neuron (LMN) or both the UMN and the LMN (mixed) [27]. When MN degeneration occurs first in the LMN, the result is flaccid dysarthria characterized by lower facial weakness, facial and tongue fasciculation and decreased tongue motility, while symptoms associated with the primary involvement of the UMN, also known as pseudobulbar palsy, are represented by spastic dysarthria with dysphagia, spastic tongue, and decreased tongue motility. Regardless of its origin, an earlier diagnosis can lead to the better management of symptoms. Nevertheless, it does not change the progression of the disease.

Communication disorders are a significant obstacle to activities and participation in social and civic life and impair quality of life. ALS patients can benefit from compensatory speech techniques, where various techniques can improve poor speech understanding and maintain communication skills for as long as possible [29].

As described in this review, specialized speech and language therapy for ALS patients with mild or moderate dysarthria is useful in maintaining communication, as is palatal elevation for velopharyngeal dysfunction [27,64,68]. Since improving lung function and respiratory muscle strength can improve dysarthria, clinical studies suggest a potential benefit of training respiratory function and respiratory muscle strength with various breathing exercises, especially non-invasive ventilation [75,79]. When non-invasive ventilation techniques are no longer effective in treating symptoms, a percutaneous dilated tracheostomy (PDT) may be performed with adjunctive therapies to treat respiratory insufficiency and, eventually, assistive devices for alternative communication [86].

Since therapeutic strategies may be more effective if the disease is diagnosed at an initial stage, early diagnosis is important not only for the possibility of being included in experimental therapies, but also because early treatment can significantly improve the quality of life of ALS patients [18]. This is particularly critical for patients with bulbar-onset ALS, where dysarthria often presents as the first symptom. As speech rate tends to decline before a definitive diagnosis is made, early therapeutic intervention can help maintain communication for a longer period [42].

The development of digital speech biomarkers and AI-based speech analysis tools has transformed our ability to detect early changes in speech. These innovations provide opportunities for more accurate and timely diagnosis, potentially well before other clinical signs become evident [50]. Evidence from Parkinson’s disease research suggests that language-based biomarkers can predict neurodegenerative disease conversion years in advance [87].

The analysis of digital speech biomarkers could represent a new frontier in the accurate characterization of the components of dysarthria in an affordable and non-invasive way and improve the possibility of interventions towards a personalized treatment approach.

## Figures and Tables

**Table 1:** Applicability of speech biomarkers in ALS patients.

Speech Dimension	Dysarthria Characteristics Compared to Healthy Speech	References
Dysprosody		
Pitch variability	Reduced	[51]
Imprecise articulation		
Vowel space area	Reduced	[52]
Consonant duration	Increased	[53]
Dysphonia		
Voice quality	Reduced	[54]
Abnormal speech timing		
Speech rate	Reduced	[55]
Pause duration	Increased	[55]
Speech impairment and fluency severity		
Dysarthria severity	Increased	[56]
Intelligibility	Reduced	[57]

## Data Availability

No new data were created or analyzed in this study. Data sharing is not applicable to this article.
